# Deciphering the role of SPL12 and AGL6 from a genetic module that functions in nodulation and root regeneration in *Medicago sativa*

**DOI:** 10.1007/s11103-022-01303-7

**Published:** 2022-08-17

**Authors:** Vida Nasrollahi, Ze-Chun Yuan, Qing Shi Mimmie Lu, Tim McDowell, Susanne E. Kohalmi, Abdelali Hannoufa

**Affiliations:** 1 Agriculture and Agri-Food Canada, 1391 Sandford Street, London, ON N5V 4T3 Canada; 2 Department of Biology, University of Western Ontario, 1151 Richmond Street, London, ON N6A 3K7 Canada

**Keywords:** Alfalfa, *SPL12*, *AGL6*, Nodulation, Root architecture, Nitrogen fixation

## Abstract

Our results show that SPL12 plays a crucial role in regulating nodule development in *Medicago sativa* L. (alfalfa), and that *AGL6* is targeted and downregulated by SPL12.

Root architecture in plants is critical because of its role in controlling nutrient cycling, water use efficiency and response to biotic and abiotic stress factors. The small RNA, microRNA156 (miR156), is highly conserved in plants, where it functions by silencing a group of SQUAMOSA-PROMOTER BINDING PROTEIN-LIKE (SPL) transcription factors. We previously showed that transgenic *Medicago sativa* (alfalfa) plants overexpressing *miR156* display increased nodulation, improved nitrogen fixation and enhanced root regenerative capacity during vegetative propagation. In alfalfa, transcripts of eleven *SPL*s, including *SPL12*, are targeted for cleavage by *miR156*. In this study, we characterized the role of SPL12 in root architecture and nodulation by investigating the transcriptomic and phenotypic changes associated with altered transcript levels of *SPL12*, and by determining SPL12 regulatory targets using *SPL12-*silencing and –overexpressing alfalfa plants. Phenotypic analyses showed that silencing of *SPL12* in alfalfa caused an increase in root regeneration, nodulation, and nitrogen fixation. In addition, *AGL6* which encodes AGAMOUS-like MADS box transcription factor, was identified as being directly targeted for silencing by SPL12, based on Next Generation Sequencing-mediated transcriptome analysis and chromatin immunoprecipitation assays. Taken together, our results suggest that SPL12 and AGL6 form a genetic module that regulates root development and nodulation in alfalfa.

**Supplementary Information:**

The online version contains supplementary material available at 10.1007/s11103-022-01303-7.

## Introduction

Alfalfa (*Medicago sativa* L.) is the most widely cultivated forage crop grown throughout the world (Annicchiarico et al. 2015). In addition to its relative tolerance to cold and drought due mainly to its deep rooting system, alfalfa’s high vegetative yield, energy value, and perennial nature make it a suitable candidate for a bioenergy crop (Sanderson and Adler 2008; Small 2010; Comas et al. 2013; Lizhen et al. 2015). While alfalfa is used mainly as a feed for livestock, it is also used for crop rotations and soil improvement because of its ability to form a symbiotic relationship with rhizobial bacteria, which improve soil nitrogen balance and quality through nitrogen fixation (Jonker and Yu 2016). The ability to fix nitrogen through these bacteria reduces the need for application of nitrogen fertilizer for alfalfa, and for crops following it in rotation (Small 2010; Blesh and Drinkwater 2013). Although alfalfa’s relationship with these bacteria is one of the most efficient relationships between rhizobial bacteria and legume plants, the amount of fixed nitrogen is variable in different planting areas and crop management systems. However, alfalfa can fix between 4 and 650 kg/ha/year of nitrogen, depending on area and environment (Peoples and Baldock 2001; Oliveira et al. 2004; Issah et al. 2020).

The symbiotic nitrogen fixation of legumes takes place in specialized organs called root nodules (Madsen et al. 2010). Nodulation is initiated by plant root exudates (phenolic flavonoid compounds) which attract bacteria to the rhizosphere and subsequently stimulate the secretion of lipo-chito-oligosaccharides, known as nod factors (NF) (Ferguson et al. 2010; Oldroyd et al. 2011), which trigger a root signaling cascade essential for rhizobia infection that has been widely studied (Oldroyd and Downie 2004; Miller et al. 2013; Yuan et al. 2017). Calcium oscillation in the nuclear region, also known as calcium spiking, is one of the earliest NF-induced responses in legume root hairs. Perception of the calcium spiking signal is deciphered by a nuclear calcium-calmodulin-dependent protein kinase known as “Does not Make Infections 3” (DMI3) in *Medicago truncatula*. *Mt*DMI3 interacts with the nuclear protein *Mt*IPD3 (Interacting Protein of DMI3) and other downstream components, such as two GRAS family proteins, Nodulation Signaling Pathway1 (NSP1) and NSP2, Nuclear Factor YA1 (NF-YA1)/YA2, ERF Required for Nodulation (ERN2), and Nodule Inception (NIN), which are essential for rhizobium infection and nodule organogenesis (Schauser et al. 1999; Kaló et al. 2005; Smit et al. 2005; Andriankaja et al. 2007; Marsh et al. 2007; Middleton et al. 2007).

Nodule development is a greatly energy-demanding process (Matsunami et al. 2004). Therefore, the host plant tightly regulates the total root nodule number to balance costs and benefits depending on the metabolic status of the shoot (carbon sink) and root (nitrogen sink) (Suzaki et al. 2015). To achieve this, legumes have evolved a negative regulatory pathway called autoregulation of nodulation (AON) to maintain an optimal number of nodules that functions systematically through the shoot (Kosslak and Bohlool 1984; Caetano-Anollés and Gresshoff 1991; Reid et al. 2011). The nitrogen regulation pathway is activated in root cortical cells during rhizobial infection and nodule development to inhibit nodulation under nitrogen‐rich conditions, helping the plant to conserve energy resources (Reid et al. 2011; Lim et al. 2014). In *M. truncatula*, root-derived nodulation-specific CLAVATA3/EMBRYO SURROUNDING REGION (CLE) peptides, including *MtCLE12* and *MtCLE13* (Mortier et al. 2010), are triggered to activate the AON following initial rhizobial infection events. These small functional CLE peptides are then translocated from root to shoot through the xylem, where they are perceived by a specific receptor complex, SUPER NUMERIC NODULES (SUNN) in *M. truncatula* (Schnabel et al. 2005). Consequence, a shoot-derived inhibitor (SDI) is produced by a still unknown signaling cascade in shoots, and moves to the roots via the phloem, where it inhibits nodule development. This regulation has been suggested to occur by reducing the activity of the transcription factor NIN (Delves et al. 1986; Lin et al. 2010; Sasaki et al. 2014; Soyano et al. 2014; Tsikou et al. 2018).

A deep rooting system is advantageous for plants, to allow access to water and nutrients stored deep in the soil, for survival under water and nutrient stress (Comas et al. 2013). In legumes, depending on the environmental conditions, root system architecture is determined predominantly by two types of lateral organs, lateral roots and nitrogen (N) fixing root nodules (Bensmihen 2015). Nodules are induced by environmental cues like low nitrogen-availability in the presence of specific *Rhizobium* spp. in the rhizosphere (Crespi and Frugier 2008; Reid et al. 2011). Formation of lateral roots is regulated by a combination of local and systemic pathways (Malamy 2005). Many factors contribute to the regulation of lateral organ formation, including mobile phytohormones (Fukaki and Tasaka 2009), microRNAs (Chen 2012) and signaling peptides (Murphy et al. 2012).

miR156 (microRNA156) regulates a range of *SPL* (*SQUAMOSA PROMOTOR BINDING PROTEIN-Like*) genes in various plant species (Jeyakumar et al. 2020). The SPL proteins constitute a diverse family of transcription factors characterized by a highly conserved SBP (SQUAMOSA-PROMOTER BINDING PROTEIN) domain, which is typically 76 amino acids long (Klein et al. 1996; Yamasaki et al. 2004; Yang et al. 2008), and is binding to a consensus DNA binding site with a NNGTACR core consensus sequence, where N is any nucleotide, and R is either A or G (Birkenbihl et al. 2005; Yamasaki et al. 2006). In alfalfa, 11 out of 22 *SPL* genes (*SPL2*, *SPL3*, *SPL4*, *SPL6*, *SPL7a*, *SPL8*, *SPL9*, *SPL11*, *SPL12*, *SPL13* and *SPL13a*) are repressed by miR156 via transcript cleavage (Aung et al. 2015; Gao et al. 2016; Feyissa et al. 2021; Ma et al. 2021; Arshad and Hannoufa 2022). Of the known SPLs in alfalfa, SPL13 has been well characterized, and has been shown to regulate flowering time and vegetative development in alfalfa, with increased lateral shoot branching in SPL13-silenced plants (Gao et al. 2018a). SPL13 also negatively regulates tolerance to drought and heat stress in this plant (Arshad et al. 2017; Matthews et al. 2019).

In Arabidopsis, *At*SPL3, *At*SPL9, and *At*SPL10 are involved in the regulation of Arabidopsis lateral root development, with *At*SPL10 playing the most dominant role (Yu et al. 2015b). Gao et al. (2018b) recently reported that *At*SPL10 directly regulates *AGAMOUS-LIKE 79* (*AGL79*) expression by binding to its promoter. *At*SPL9 is demonstrated to be a potential nitrate regulatory hub and may target the primary nitrate-responsive genes (Krouk et al. 2010). It has been shown that transcript levels of nitrate-responsive genes, *nitrite reductase* (*NiR*), *nitrate reductase* (*NIA2*) and a high-affinity nitrate transporter gene (*NRT1*.*1*) significantly increased in response to nitrate in *AtSPL9* overexpressing transgenic Arabidopsis plants (Krouk et al. 2010).

The MADS (MCM1/AGAMOUS/DEFICIENS/SRF) box proteins are a family of transcription factors that participate in many aspects of plant development and morphogenesis (Gramzow and Theissen 2010). Although MADS-box proteins were initially found to be involved in floral organ speciation (Michaels et al. 2003; De Folter et al. 2006; Dong et al. 2013; Huang et al. 2017), they recently became a focus of research into the genetic regulation of root development (reviewed by Alvarez‐Buylla et al. 2019). For example, *At*ANR1 (Arabidopsis NITRATE REGULATED1), was the first MADS-box TF identified to stimulate lateral root development in the presence of high nitrate concentrations (Gan et al. 2012). *AtAGL21*, which is highly expressed in lateral root primordia, and was found to be a very important regulator of lateral root development by regulating auxin biosynthesis genes in Arabidopsis (Yu et al. 2014). In rice, *OsMADS25*, an *ANR1*-like gene, positively regulates lateral and primary root development by promoting nitrate accumulation and increasing the expressions of nitrate transporter genes at high nitrate concentration (Yu et al. 2015a).

In this study, we used overexpression (OE) and RNAi silencing to conduct a functional characterization of SPL12 in alfalfa. Through phenotypic analysis of both *35S::SPL12* and *SPL12*-RNAi plants, possible roles of SPL12 in root regeneration, nodulation and nitrogen fixation were explored. We used Next Generation Sequencing (NGS)-based transcriptome analysis of *SPL12*-RNAi plants to identify possible downstream genes that may be directly targeted for regulation by SPL12. This study links SPL12 to specific traits like nodulation and root architecture in alfalfa and to the *AGL6* gene, and sheds light on the miR156-SPL regulatory network in alfalfa.

## Materials and methods

### Plant material and growth conditions

*M. sativa* L. (alfalfa) clone N4.4.2 (Badhan et al. 2014) was obtained from Daniel Brown (Agriculture and Agri-Food Canada, London, ON, Canada) and was used as wild-type (WT) genotype. Plants overexpressing miR156 (miR156OE) at different levels (A11, A11a and A17) were obtained from our previous study (Aung et al. 2015). WT and transgenic alfalfa plants were grown under greenhouse conditions at 21–23 °C, 16-h light/8-h dark per day, light intensity of 380–450 W/m2 (approximately 500 W/m2 at high noon time), and a relative humidity of 56% for the duration of all experiments. Because of the obligate outcrossing nature of alfalfa, WT and transgenic alfalfa were propagated by rooted stem cuttings to maintain the genotype throughout the study. Stem cutting propagation and morphological characterization of alfalfa plants were carried out as described in Aung et al. (2015).

### Generation of vector constructs and plant transformation

*35S::SPL12* (L1, L5, and L7), *SPL12*-RNAi (RNAi12-7, RNAi12-24 and RNAi12-S29), and *AGL6*-RNAi (L9, L13A and L13B) genotypes were generated to investigate the role of SPL12 and AGL6 in root architecture and nodulation. For *SPL12*-RNAi and *AGL6*-RNAi, 250 and 256 bp fragments, respectively, were amplified from alfalfa cDNA using RNAiMsSPL12-F2 and RNAiMsSPL12-R2 (*SPL12*-RNAi), and MsAGL6-RNAi-F2 and MsAGL6-RNAi-R2 (*AGL6*-RNAi) (Table S1) primers and cloned into pENTR entry vector (Invitrogen, Carlsbad, CA, USA). After PCR screening and confirmation by sequencing, LR reactions were performed for RNAi constructs to recombine the fragments into the pHELLSGATE12 (RNAi) destination vector (Helliwell and Waterhouse 2003) using the Gateway cloning system (Thermo Fisher Scientific, Mississauga ON).

To generate *SPL12* overexpression constructs, the full-length coding fragment of *SPL12* (1314 bp) was amplified from alfalfa cDNA using primers OEMsSPL12 F and OEMsSPL12 R (Table S1), which was cloned downstream of *CaMV35S* (*35S*) promoter to generate *35S::SPL12* construct. The fragments were then cloned into the pMDC32 vector using Gateway cloning. For *35S:SPL12m-GFP* construct, the *Mlu*I-SPL12-*Spe*I fragment was synthesized with a mutated miR156 recognition site based on Wei et al. (2012). The fragments were then cloned into pGreen-GFP vector using a T4 ligation method according to the manufacturer’s description (Thermo Fisher Scientific).

Subsequently, overexpression and RNAi constructs were transformed into *Agrobacterium tumefaciens* LBA4404 or EHA105 by electroporation or heat shock, respectively. *A. tumefaciens* strains were then used to transform alfalfa clone N4.4.2 by a tissue culture-based method (Aung et al. 2015). The presence of the transgenes in *SPL12*-RNAi and *AGL6*-RNAi alfalfa genotypes were confirmed by PCR using gDNA as the template and using a *35S* promoter and pHellgate12 intron primers (pHG12int R2) (Table S1). Similarly, *SPL12* overexpression alfalfa genotypes (*35S::SPL12* and *35S:SPL12m-GFP*) were screened by PCR using a *35S* promoter and gene-specific primers (OEMsSPL12-R) (Table S1). Positive transgenic plants were then analyzed for *SPL12* and *AGL6* transcript abundance by RT-qPCR using primer pairs LA-MsSPL12-Fq1 and LA-MsSPL12-Rq1, and qMsAGL6-1F and qMsAGL6-1R, respectively (Table S1).

### Phenotypic analysis of root and nodule development

Root development from the stems was determined for transgenic and WT plants grown in vermiculite at 13 days after initiation of vegetative propagules as described in Aung et al. (2017).

To determine the number of nodules, plants were examined at 14 and 21 days after inoculation (dai) with *Sinorhizobium meliloti* Sm1021. To eliminate potential microbial contamination, equipment was surface-sterilized using 1% sodium hypochlorite, while vermiculite and water were sterilized by autoclaving for 1 h. *S. meliloti* Sm1021 strain was cultured on a Yeast Extract Broth agar for two days at 28 °C. A single colony was then inoculated in liquid TY medium and incubated at 28 °C to an optical density OD_600_ nm of 1.5. The alfalfa rooted stems were inoculated by applying 5 mL of bacterial suspension or sterilized water (non-inoculated control) as described in Aung et al. (2015). Nodule phenotypes were recorded by photography under a stereo microscope (Nikon SMZ1500, Japan) using 1-mm magnification.

Nitrogenase activity was determined in nodulated roots at 14 dai by the acetylene reduction assay as described in Aung et al. (2017). The amount of ethylene released from acetylene reduction was then calculated and expressed as root nmol/plant.

### Analysis of SPL12-GFP fusion proteins by Western blotting

Fresh leaves of 30-day-old plants of WT and *35S:SPL12m-GFP* alfalfa were homogenized in 0.2 ml of protein extraction buffer (0.125 mM Tris, pH 6.8, 4% w/v SDS, 18% glycerol, 0.024% w/v bromophenol-blue, 1.43 M β-mercaptoethanol, 0.2% protease inhibitor). After centrifugation at 15,000 g for 10 min the insoluble fraction (pellet) was discarded, and the denatured proteins were separated on a 12% SDS PAGE gel. Separated proteins were then transferred onto a nitrocellulose membrane, followed by incubation with primary anti-GFP antibody (Abcam, ab290, Cambridge, MA) and secondary horseradish peroxidase (HRP)-conjugated goat antirabbit IgG (Abcam) antibody. The signals were developed using Pierce ECL Western Blotting Substrate (Thermo Fisher Scientific, Waltham, MA).

### RNA extraction, reverse transcription and RT‑qPCR

Different alfalfa tissues (stems, leaves and roots) were collected and flash frozen in liquid nitrogen and stored at -80 °C until further use. Approximately 100 mg fresh weight was used for total RNA extraction using RNeasy Plant Mini-prep Kit (Qiagen, Cat # 1708891) for leaf and stem samples, and Total RNA Purification Kit (Norgen Biotek, Cat # 25800) for roots. Tissue was homogenized using a PowerLyzer®24 bench top bead-based homogenizer (Cat # 13155) according to the manufacturer’s manual. Approximately 500 ng of Turbo DNase (Invitrogen, Cat # AM1907)-treated RNA was used to generate cDNA using the iScript cDNA synthesis kit (Bio-Rad, Cat # 1708891). Transcript levels were analyzed by RT-qPCR using a CFX96 TouchTM Real-Time PCR Detection System (Bio-Rad) and SsoFast™ EvaGreen® Supermixes (Bio-Rad Cat # 1725204) using gene specific primers. Each reaction consisted of 2 μL of cDNA template, 0.5 μL forward and reverse gene-specific primers (10 μM each) (Table S1), 5 μL SsoFast Eva green Supermix and topped up to 10 μL with ddH_2_O. For each sample three or four biological replicates were analyzed, and each biological replicate was tested using three technical replicates. Transcript levels were analyzed relative to three reference genes: *CYCLOPHILIN* (Cyclo) (Guerriero et al. 2014), *β-actin* (*ACTB*) (Castonguay et al. 2015) and *ACTIN DEPOLYMERIZING FACTOR* (*ADF*) (Guerriero et al. 2014; Castonguay et al. 2015) (primers are listed in Table S1).

### Next generation RNA sequencing transcriptome analysis

About 5 cm of root tips from WT and two *SPL12*-RNAi genotypes (RNAi12-24 and RNAi12-29) were used for Next Generation RNA sequencing. Total RNA was extracted using the RNeasy PowerPlant Kit (Qiagen, Cat # 13,500–50) and quantified using a NanoDrop 2000C (Thermo Scientific). RNA quality was assessed with Agilent Bioanalyzer 2100 RNA Nano chip (Agilent Technologies). There were three biological replicates for each genotype. An RNA library was constructed and sequenced on an Illumina NovaSeq6000 with 100 bp fragment pair end reads at Genome Quebec (Montreal, Canada) as a fee-for-service.

### Analysis of differentially expressed genes (DEGs) and GO enrichment analysis

RNAseq data were analyzed using published protocols (Trapnell et al. 2012) on Biocluster with Linux shell scripts. *M. truncatula* Mt4.0 V2 (http://www.medicagogenome.org/downloads) was used as a reference genome. Firstly, the QC analyses were performed for all Raw Illumina pair-end reads using FastQC program. Raw sequence reads were then trimmed to obtain high quality reads (*Q* > 30), adapter sequences were removed, and short reads dropped using custom Perl scripts. These high-quality reads were then mapped to the *M. truncatula* genome using TopHat (v2.0.10). Tophat output was then used as input files for Cufflink (v2.2.1) to detect differentially expressed genes between WT and *SPL12*-RNAi (Aung et al. 2017). Subsequently, differentially expressed genes were annotated and assigned to three major functional categories (biological process, molecular function, cell component) using Reduced Visualization Gene Ontology (REVIGO) software (http://revigo.irb.hr/) as described in Supek et al. (2011). Venn diagrams were generated using the Venny tool (Oliveros 2007). RNA-seq raw data can be accessed from the National Center for Biotechnology Information, NCBI, BioProject PRJNA818300.

### ChIP‑qPCR analysis

Shoot tips of alfalfa plants overexpressing *SPL12* tagged with *GFP* driven by the *35S* promoter (*35S:SPL12m-GFP*) were used as materials for ChIP-qPCR analysis, which was performed based on a previously described protocol (Gendrel et al. 2005), with minor modifications, using the Chromatin Immunoprecipitation Assay kit (Lot:2,382,621, Millipore, Billerica, MS). Briefly, 1 g of shoot tips from WT and *35S:SPL12m-GFP* plants were collected and fixed with 1% formaldehyde under vacuum for 20 min. The reaction was stopped by adding 0.125 M glycine, and the fixed tissues were ground in liquid nitrogen. Powdered tissues were homogenized with 30 ml of pre-chilled Extraction Buffer 1 (Extraction reagents and buffers are listed in Table S2) and incubated for 10 min on ice, then the crude extract was filtered with two layers of Miracloth (Millipore, Canada). The filtrate was centrifuged at 3000 g for 20 min and the supernatant was discarded while the pellets were re-suspended in 1 ml of pre-chilled Extraction Buffer 2. After centrifugation at 12,000 *g* for 10 min, the pellets were re-suspended in 300 μL pre-chilled Extraction Buffer 3 and centrifuged at 16,000 *g* for 1 h. The supernatant was removed, and chromatin pellets were re-suspended in 300 μL of Nuclei Lysis Buffer by gentle pipetting. The chromatin solution was then sonicated twice at power 3 for 15 s on ice into 500–1000 bp fragments using a Sonic Dismembrator (Fisher Scientific). A 15 μL aliquot of the supernatant was removed to use as the Input DNA control. A total of 30 μL of protein A-agarose beads (Millipore, Canada) was added to the Chromatin solution that was brought to 1.5 mL using ChIP dilution buffer, and this mixture was rotated for 1 h at 4 °C. Subsequently, the mixture was gently agitated, centrifuged (3500 g) for 1 min, and the supernatant was transferred for immunoprecipitation while discarding the beads. A total of 5 μL of Ab290 GFP antibody was added to the chromatin solution and the mixture was incubated with overnight gentle agitation at 4 °C. After 12 h, 50 μL of protein A-agarose beads were added to each tube and immune complexes were collected by incubation at 4 °C for at least 1 h with gentle agitation and then centrifugation. After washing with a cycle of low normality salt, high salt, LiCl and TE (Tris–EDTA) buffer, the immunoprecipitate was eluted with 250 μL of Elution Buffer. The DNA reverse cross-linking procedure was performed with 20 μL of 5 M NaCl incubated at 65 °C for 5 h. To each sample 10 μL 0.5 M EDTA, 20 μL 1 M Tris–HCl (pH 6.5) and 2 μL of 10 mg/mL proteinase K (Sigma- Aldrich, Canada) was added. DNA was extracted using phenol: chloroform (1:1, v:v), recovered by ethanol precipitation in the presence of 0.3 M sodium acetate (pH = 5.2) and 2 μL glycogen carrier 10 mg/mL (Sigma-Aldrich, Canada), after overnight incubation at – 20 °C. The DNA pellets was washed with 70% ethanol and each pellet was re-suspended in 16 μL of distilled water to be used for ChIP-qPCR analysis using qnMsAGL6 (Table S1). SPL12 occupancy on *AGL6* was estimated by comparing the fold enrichment in *35S:SPL12m-GFP* and WT plants. A DNA fragment containing a SBP binding consensus-like sequence was amplified from *LATERAL ORGAN BOUNDARES-1* (*LOB1*) (Shuai et al. 2002) and used as a negative control.

### Statistical analysis

Statistical analyses were performed using Microsoft Excel spread-sheet software. Pairwise comparisons were made using a Student’s t-test with either equal or unequal variance. The significant differences between sample means for three or more data sets were calculated using the one-way analysis of variance (ANOVA) where appropriate.

## Results

### Analysis of *SPL12* transcript levels in *SPL12*-RNAi and *35S::SPL12* plants

*SPL12* overexpression plants (*35S::SPL12*), *SPL12*-RNAi plants, and wild-type (non-transformed) plants (WT) were used to study the role of SPL12 in various root traits in alfalfa by analyzing first the relative levels of *SPL12* transcript. *35S::SPL12* genotypes, L1, L5, and L7, were found to overexpress *SPL12* relative to WT plants (Fig. S1A).

As *SPL12* is one of the *SPL* genes that are silenced by miR156 in alfalfa (Aung et al. 2015; Gao et al. 2016), we generated RNAi-silenced *SPL12* (*SPL12*-RNAi) plants. Of the 33 plants harboring the *SPL12*-RNAi construct, we chose three genotypes (RNAi12-7, RNAi12-24, and RNAi12-29) with the lowest (43%, 36% and 32% of WT) *SPL12* transcript levels (Fig. S1B) for subsequent analyses.

### Effect of *SPL12* silencing on root regenerative capacity

**Fig. 1:**
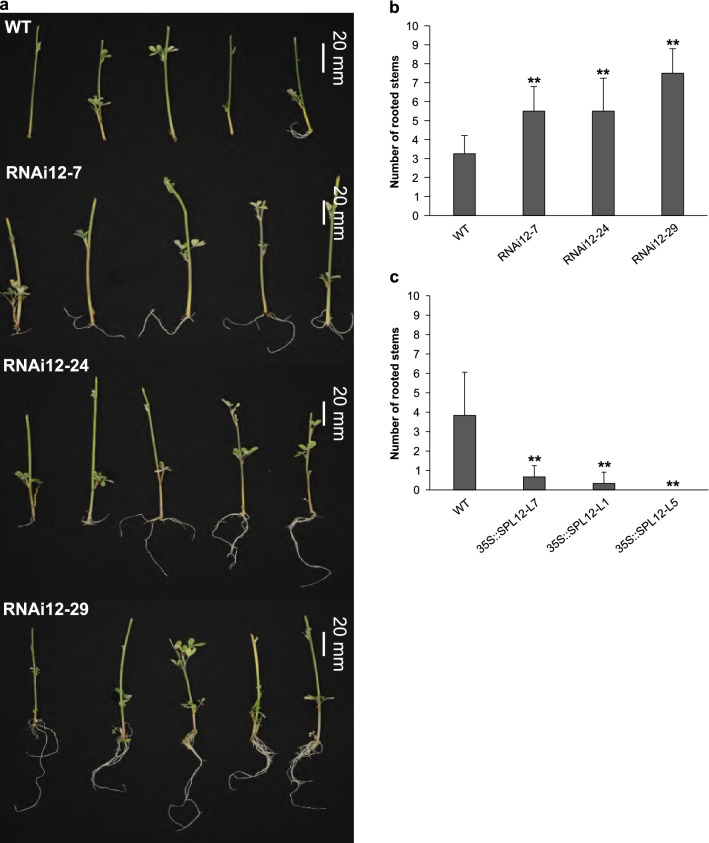
Effect of *SPL12* silencing and overexpression on root development in alfalfa. **A** Typical root regeneration phenotype from stem cuttings at 13 days after vegetative propagation. **B** Number of rooting stems arising from 14 stems (per replicate) at 13 days after vegetative propagation in WT, and the *SPL12*-RNAi genotypes (n = 14). **C** Number of rooting stems arising from 12 stems (per replicate) at 13 days after vegetative propagation in WT, and the *35S::SPL12* genotypes (n = 12). * and ** indicate significant differences relative to WT using t test p < 0.05, p < 0.01, respectively. Error bar indicates standard deviation

Transgenic *SPL12*-RNAi genotypes and WT alfalfa were vegetatively propagated by stem cuttings to assess root regeneration capacity. As early as 10 days after vegetative propagation, root regeneration from stem nodes could be observed in one or more of the *SPL12*-RNAi genotypes. Compared to WT plants, the number of rooted stem propagules was significantly higher in *SPL12*-RNAi transgenic alfalfa genotypes at 13 days post propagation (Fig. 1A, B). Genotype RNAi12-29 showed an increase in root regeneration capacity earlier than others (data not shown), but genotypes RNAi12-7 and RNAi12-24 still showed a higher rooting compared to WT (Fig. 1B). To further investigate the role of SPL12 in root regeneration we analyzed this trait in transgenic alfalfa plants overexpressing *SPL12*. The number of rooted stem propagules was decreased by more than 5.75-fold in *35S::SPL12* plants compared to WT control (Fig. 1C).

### Effect of inoculation with *S. meliloti* on *SPL12* transcript levels

**Fig. 2:**
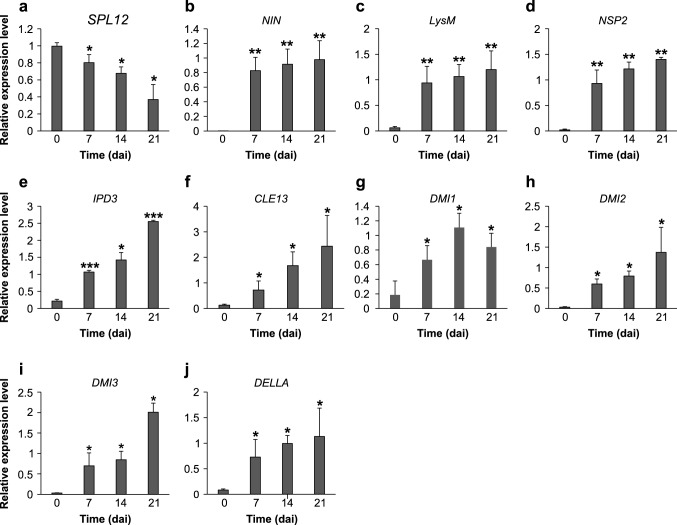
Transcript analysis of *SPL12* and early nodulation genes upon rhizobial infection. Transcript levels of *SPL12* (**A**), and early nodulation genes (**B**–**J**) were determined in roots inoculated with *S. meliloti* at 0, 7, 14 and 21 days after inoculation (dai). Seven of the alfalfa early nodulation genes including: *NIN* (**B**), *LysM* (**C**), *NSP2* (**D**), *IPD3* (**E**), *CLE13* (**F**), *DMI1* (**G**), *DMI2* (**H**), *DMI3* (**I**), and *DELLA* (**J**). *, ** and *** indicate significant differences relative to wild type using t test (n = 3) p < 0.05, p < 0.01, and p < 0.001, respectively. Error bar indicates standard deviation

To gain an insight into the role of *SPL12* in the alfalfa-*S. meliloti* symbiosis, we determined its transcript levels in inoculated roots of wild type alfalfa (WT) (Fig. 2). To analyze *SPL12* regulation at different stages of the symbiotic process, rooted WT alfalfa plants (14 days after cutting) were inoculated with *S. meliloti* Sm1021 and transcript analysis was carried out at 0, 7, 14 and 21 dai. The relative transcript levels of *SPL12* decreased gradually, with the lowest transcript detected at 21 dai (Fig. 2A).

To investigate if *SPL12* transcript levels correlate with relevant events in the rhizobial infection process, we analyzed the transcript levels of some of early nodulation genes in inoculated roots (Fig. 2B–J). These genes include *NIN* (Marsh et al. 2007), *NSP2* (Kaló et al. 2005), *IPD3* (Messinese et al. 2007), *DMI1* (Ané et al. 2004), *DMI2* (Bersoult et al. 2005), *DMI3* (Messinese et al. 2007), *DELLA* (Jin et al. 2016), *LysM* (Arrighi et al. 2006), and *CLE13* (Mortier et al. 2010). The transcript levels of all the genes increased significantly compare to 0 dai (Fig. 2B–J), indicating a possible function for *SPL12* in nodulation.

### SPL12 regulates nodulation

**Fig. 3:**
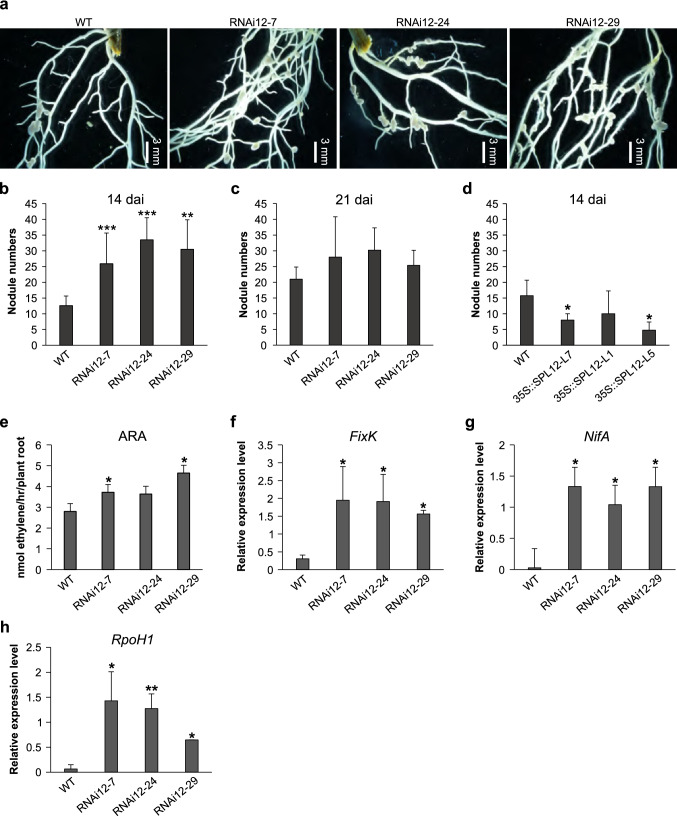
The effect of the *SPL12* silencing and overexpression on nodulation and nitrogen fixation. **A** Nodule phenotypes of WT, and the *SPL12*-RNAi genotypes at 14 dai. **B** The number of nodules in WT and the *SPL12*-RNAi at 14 dai, and **C** at 21 dai (n = 13–15 plants). **D** The number of nodules in WT and the *35S::SPL12* at 14 dai, and (n = 9–12 plants). **E** Nitrogenase activity (Acetylene reduction activity; nmol ethylene/hr/plant root) in transgenic alfalfa at two weeks after inoculation with *S. meliloti* (n = 5 plants). **F**, **G**, **H** Transcript levels for *S. meliloti* **F** *FixK*, **G** *NifA*, and **H** *RpoH* genes in alfalfa roots inoculated with rhizobia. Transcript levels was analyzed using three biological replicates and two technical replicates. * and ** indicate significant differences relative to wild type using *t* test (n = 3) p < 0.05, p < 0.01, respectively. Error bar indicates standard deviation

Overexpression of miR156 was reported earlier to increase root length and enhance nodulation in transgenic alfalfa genotypes (Aung et al. 2015), so we investigated the root phenotypes in WT and *SPL12*-RNAi. To determine the ability of *SPL12*-RNAi transgenic rooted stems to form symbiotic nodules, two weeks post cutting, the rooted transgenic plants were inoculated with *S. meliloti* 14 dai and 21 dai. At 14 dai, *SPL12* silencing increased nodulation by more than 2.1-fold in RNAi12-7, RNAi12-24 and RNAi12-29 compared to WT plants (Fig. 3A, B), however, at 21 dai no significant difference between *SPL12*-RNAi genotypes and WT was observed (Fig. 3C).

To determine the ability of *35S::SPL12* transgenic plants to form symbiotic nodules, 2 weeks after cutting, the rooted transgenic plants were inoculated with *S. meliloti* for 14 days. Among the *35S::SPL12* genotypes the total nodule number was significantly decreased in L7, L5 compared with the WT control at 14 dai (Fig. 3D). These results suggest that the transcript levels of *SPL12* is negatively correlated to nodulation and root regeneration in alfalfa.

### Silencing of *SPL12* enhances nitrogen fixation

To investigate the role of SPL12 in alfalfa nitrogen fixation, we analyzed the effect of *SPL12* silencing on nitrogenase activity. Two-week-old *SPL12*-RNAi plants were inoculated with *S. meliloti* and allowed to grow in the absence of nitrate for an additional two weeks. During this time the mature nodules formed, and a significant increase in nodulation was observed in RNAi12-7, RNAi12-24 and RNAi12-29 genotypes relative to WT (Fig. 3B). The nitrogenase activity in the nodule was determined by acetylene reduction activity (ARA). The ARA of the nodulated roots of transgenic alfalfa genotypes RNAi12-7 and RNAi12-29 was significantly increased compared to that of WT plants (Fig. 3E). The level of ethylene production was the highest from roots of genotype RNAi12-29 (4.64 nmol/plant) whereas the WT control plant contained the lowest level of ethylene (2.8 nmol/plant). Furthermore, given the increased nitrogenase activity of nodules in the *SPL12*-RNAi genotypes, the transcript levels of several bacterial genes including *FixK* (providing activation of nodule respiration), *NifA* (nitrogenase-encoding) and *RpoH* (sigma 32 factor for effective nodulation) in alfalfa roots inoculated with *S. meliloti* were also investigated. Compared to WT, *SPL12*-RNAi showed increased transcript levels of *NifA*, *FixK* and *RpoH* genes (Fig. 3F–H). These findings suggest that *SPL12* silencing enhances both nodulation and nitrogen fixation in alfalfa.

### *SPL12* silencing affects nodulation-related genes

**Fig. 4:**
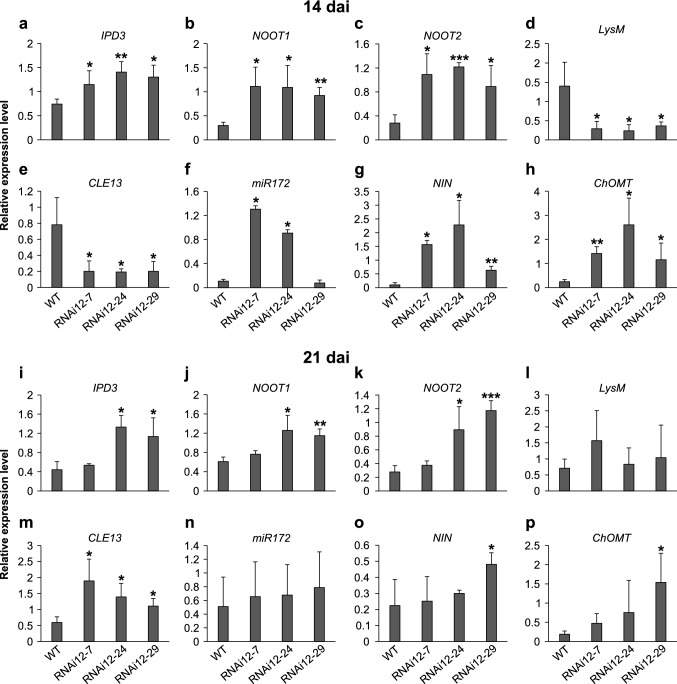
Transcript analysis of nodulation-related genes in *SPL12*-RNAi genotypes. Relative transcript levels at 14 dai (**A**–**H**) and at 21 dai (**I**–**P**) for **A** *IPD3,* **B** *NOOT1,* **C** *NOOT2,* **D** *LysM,* **E** *CLE13,* **F** *miR172,* **G** *NIN,* **H** *ChOMT,* **I** *IPD3,* **J** *NOOT1*, **K** *NOOT2,* **L** *LysM,* **M** *CLE13,* **N** *miR172,* **O** *NIN,* and **P** *ChOMT*. *, ** and *** indicate significant differences relative to wild type using *t* test (n = 3) p < 0.05, p < 0.01, and p < 0.001, respectively. Error bar indicates standard deviation

Given the above finding that *SPL12*-RNAi alfalfa plants have enhanced nodulation (at 14 dai), we examined transcript levels of several nodulation-related genes at 14 dai and at 21 dai in alfalfa plants. We found that *SPL12* silencing differentially regulated the transcript levels of *IPD3* (Messinese et al. 2007), *LysM* (Arrighi et al. 2006), *NOOT1*, *NOOT2* (Magne et al. 2018)*, CLE13* (Mortier et al. 2010)*, miR172* (Gao et al. 2016; Wang et al. 2019), *NIN* (Marsh et al. 2007), and *ChOMT* (Maxwell et al. 1992; Breakspear et al. 2014) genes in roots of alfalfa at 14 dai and 21 dai (Fig. 4). Of the tested genes, *IPD3*, *NOOT1* and *NOOT2* were significantly upregulated in all the *SPL12*-RNAi genotypes (RNAi12-7, RNAi12-24 and RNAi12-29) at 14 dai (Fig. 4A–C), but these genes were upregulated in only two of them (RNAi12-24 and RNAi12-29) at 21 dai (Fig. 4I–K). *LysM* is downregulated in all of *SPL12*-RNAi plants at 14 dai (Fig. 4D), but no significant changes were observed at 21 dai (Fig. 4L). Consistent with the increased number of nodules at 14 dai and no change at 21 dai, *SPL12*-RNAi plants at 14 dai showed reduced transcript levels of *CLE13* (Fig. 4E) with enhanced transcript levels of *miR172* in only two of *SPL12*-RNAi plants (RNAi12-7 and RNAi12-24) (Fig. 4F). However, at 21 dai, *CLE13* was significantly upregulated in the three *SPL12*-RNAi plants, whereas *miR172* did not show any significant difference (Fig. 4M, N). Moreover, significant effects of *SPL12* silencing on *NIN* and *ChOMT* transcript levels were observed in all of the *SPL12*-RNAi roots at 14 dai (Fig. 4G,H), but were upregulated in only RNAi12-29 genotype at 21 dai (Fig. 4O-P). These findings suggest the involvement of SPL12 in AON in alfalfa symbiosis.

### Effect of *SPL12* silencing on the root transcriptome

Given the potential role of SPL12 in enhancing nodulation and root emergence capacity, we carried out Next Generation Sequencing (NGS)-based transcriptomic analysis (RNA-Seq) on the root tissues of WT and *SPL12-*RNAi (RNAi12-24 and RNAi12-29) alfalfa plants to identify genes that may be regulated by SPL12. To validate the findings of the RNA-Seq data, a total of 14 genes (including upregulated and downregulated) were randomly selected and analyzed by RT-qPCR (Table S3). A total of 13 of the 14 transcripts (92%) showed similar trends of transcript levels change (Table S3), suggesting that our RNA-Seq results were reliable.

**Fig. 5:**
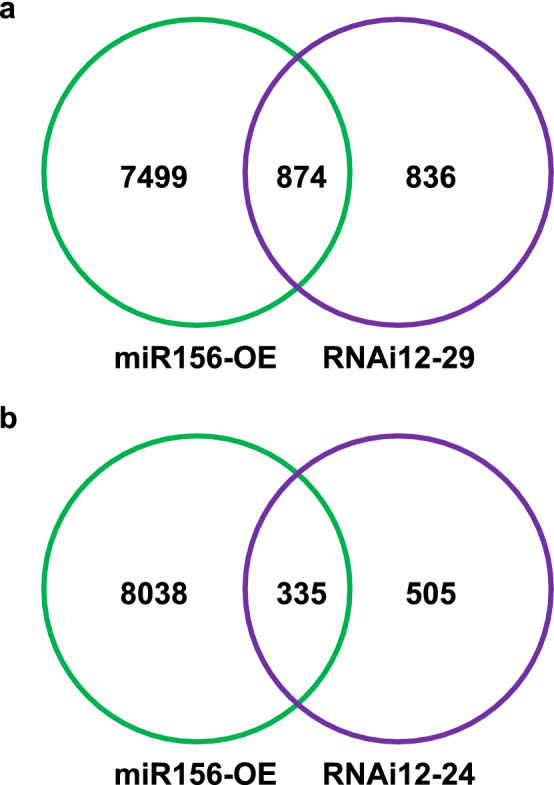
NGS-based transcriptome analysis of WT and *SPL12*-RNAi alfalfa plants. Total number of differentially expressed genes detected between **A** miR156OE and RNAi12-29, and **B** miR156OE and RNAi12-24 via NGS based transcriptome analysis

Compared to WT, a total of 1710 and 840 (p < 0.005) differentially expressed genes (DEGs) were found in RNAi12-29 and RNAi12-24 genotypes, respectively (Table S4). Previous transcriptomic analysis of miR156OE plant A17 (Aung et al. 2017) revealed 8373 differentially expressed genes when comparing WT and miR156OE roots. A comparison of the published transcriptome data of miR156OE, and each of the *SPL12*-RNAi plants, RNAi12-29 and RNAi12-24, showed that they shared 874 and 335 DEGs, respectively (Fig. 5A, B; Table S5). NGS analysis revealed that a gene encoding an AGAMOUS-like 6 (AGL6) (MS.gene052964, MS.gene071001 and MS.gene34431), a yet to be described MADS box protein was significantly downregulating in A17 (Aung et al. 2017). This gene is closely related to the Arabidopsis *AtAGL79* gene that is regulated by *At*SPL10. In Arabidopsis miR156/SPL10 regulatory pathway targets *AtAGL79* to regulate plant lateral root development (Gao et al. 2018a, b).

Gene ontology (GO) enrichment analysis of DEGs was carried out and categorized into molecular function, biological process, and cellular components to identify pathways that may be affected in *SPL12*-RNAi plants. GO-term analysis showed that 65% of DEGs are associated with molecular function followed by 26 and 9% to biological process and cellular components, respectively (Fig. S2A). Graphical representation of the components of GO-term analysis is provided in supplementary file Fig. S2B-D. In molecular function category, Catalytic activity, Binding, Hydrolase activity, Nucleotide binding, Metal ion binding, and Oxidoreductase activity are the top highly represented GO-terms (Table S6). On the other hand, among the 14 functions classified as biological processes, metabolic process, primary metabolic process, cellular biosynthetic process, and cellular aromatic compound metabolic process are the major representation of GO-terms (Table S6). The full list of the components for the three fractions (molecular function, cellular component, and biological process) is shown in Table S6.

### Transcript patterns of the *SPL12*, *AGL6* genes in alfalfa

**Fig. 6:**
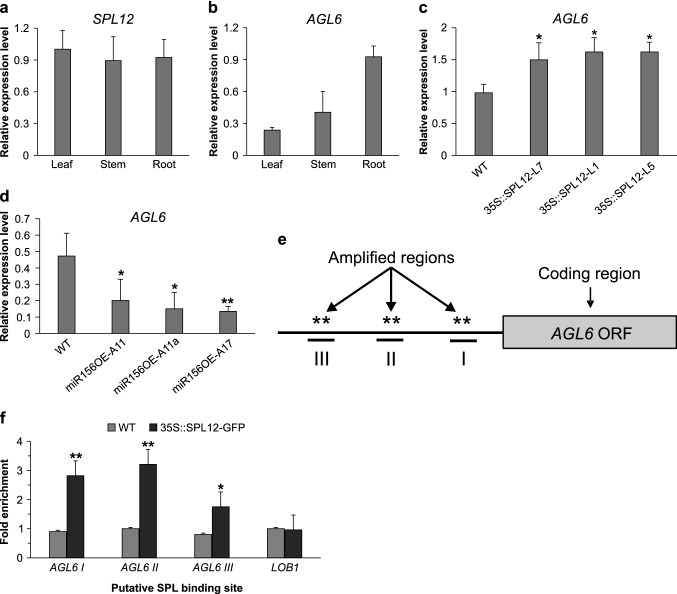
Tissue-specific transcript profiles of *SPL12*, *AGL6*, and *AGL21* genes in alfalfa. Relative gene transcript levels of *SPL12* (n = 3) (**A**), *AGL6* (n = 3) (**B**) was determined by RT-qPCR. *AGL6* transcript analysis in *35S::SPL12* (**C**) and miR156OE (**D**) relative to WT by RT-qPCR. **E** Schematic representation of the *AGL6* promoter region black box coding sequences; asterisks indicate locations of putative SPL binding sites in the *AGL6* promoter. Roman numerals (I, II and III): sites that were tested for qPCR. **F** ChIP-qPCR based fold enrichment analysis of SPL12 in SPL12m-GFP and WT plants. Shown are the means of n = three individual plants. *LATERAL ORGAN BOUNDARES-1, LOB1*, was used as a negative control. Transcript levels was analyzed using three biological replicates and three technical replicates. * and ** indicate significant differences relative to wild type using *t* test p < 0.05, p < 0.01, respectively. Error bar indicates standard deviation

To investigate the transcript profiles of *SPL12* and *AGL6* in alfalfa, we measured their transcript levels by RT-qPCR in three tissues of 21-day-old of WT alfalfa plants (leaf, stem, and root). The transcript levels of *SPL12* were detected at similar levels in all three tissues (Fig. 6A). The transcript levels of *AGL6* were also detected in the aforementioned tissues (Fig. 6B), with roots showing the highest and leaves the lower transcript levels. In roots, *AGL6* levels were higher in *SPL12* overexpressing genotypes (Fig. 6C), and lower in miR156OE genotypes (A11 and A11a and A17) compared to WT (Fig. 6D), suggesting that *AGL6* is positively regulated by SPL12.

### SPL12 is a direct regulator of *AGL6*

As *AGL6* was significantly upregulated in *35S::SPL12* (L7, L1 and L5) plants (Fig. 6C), and was downregulated in miR156OE alfalfa (Fig. 6D), we conducted further characterization using ChIP-qPCR to determine if *AGL6* is a direct target of SPL12. For that, we characterized transgenic plants expressing the SPL12m-GFP fusion protein (*35S:SPL12m-GFP*) by Western blot analysis to detect the SPL12m-GFP fusion protein (Fig. S3). There are at least five core GTAC sequences (as potential SPL12 binding sites) within 2000 bp upstream of the translation start codon of *AGL6* that are distributed in three regions (I, II, and III) (Fig. 6E; Fig. S4). These three regions were selected to test for SPL12 occupancy. A relatively strong binding capacity of SPL12 to the *AGL6* promoter region was detected by ChIP-qPCR in the *35S:SPL12m-GFP* transgenic alfalfa plants (Fig. 6F). Occupancy in these three regions was substantially higher than that in the WT and *LOB1* controls, indicating that SPL12 protein could bind directly to multiple regions in *AGL6* promoter to regulate its expression.

### AGL6 silencing enhances nodulation

**Fig. 7:**
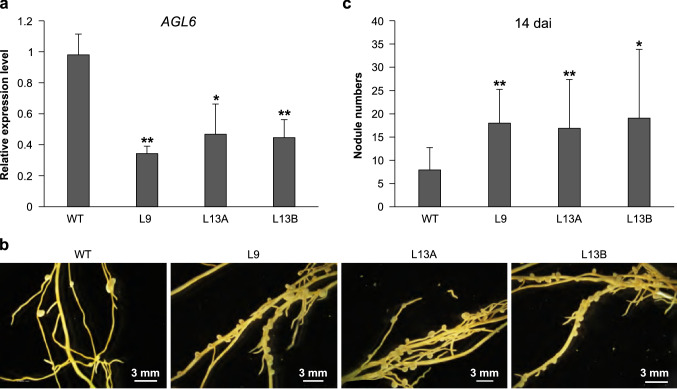
The effect of *AGL6* silencing on nodulation. **A** Relative *AGL6* gene transcript levels in *AGL6*-RNAi plants (n = 3). **B** Nodule phenotypes of WT, and the *AGL6*-RNAi genotypes at 14 dai. **C** The number of nodules in WT and the *AGL6*-RNAi at 14 dai (n = 12 plants). * and ** indicate significant differences relative to wild type using *t* test p < 0.05, p < 0.01, respectively. Error bar indicates standard deviation

To further investigate the role of *AGL6* in alfalfa nodule development, *AGL6*-RNAi transgenic plants were generated. Of the 19 plants harboring the *AGL6*-RNAi construct, we chose three genotypes (L9, L13A and L13B) that exhibited the lowest *AGL6* transcript levels (Fig. 7A) for phenotypic comparison. At 14 dai, the three *AGL6*-RNAi plants had higher number of nodules compared to WT (Fig. 7B, C), thus substantiating the finding of the likely involvement of *AGL6* in regulating nodulation in alfalfa.

## Discussion

To study the role of miR156 in alfalfa growth and development, two genotypes, A11a and A17, that overexpress miR156 were generated in a previous study. These alfalfa genotypes displayed increased nodulation, improved nitrogen fixation and enhanced root regenerative capacity during vegetative propagation (Aung et al. 2015). It was also reported that miR156 targets at least eleven *SPL* genes, including *SPL12*, for silencing by transcript cleavage (Gao et al. 2016; Feyissa et al. 2021). Whereas the role of some of the targeted SPL transcription factors, such as SPL13, SPL9, SPL8 and SPL20 have been characterized in alfalfa (Arshad et al. 2017; Gao et al. 2018a; Feyissa et al. 2019, 2021; Matthews et al. 2019; Hanly et al. 2020; Ma et al. 2021; Singer et al. 2021), the specific functions of SPL12 remain elusive, as no studies have been conducted on the possible role of miR156/SPL12 module in the development of underground tissues. In the present study, we analyzed transgenic plants with altered transcript levels of *SPL12* and *AGL6*, including *SPL12*-RNAi, *35S::SPL12*, GFP-tagged SPL12 and *AGL6*-RNAi to investigate the role of SPL12 in root architecture.

One of the major goals of our alfalfa research was to identify which SPL genes function downstream of miR156 to modulate root regeneration and nodulation in alfalfa. In Arabidopsis, it was suggested that at least one group of *At*SPLs, (*At*SPL3, *At*SPL9, and *At*SPL10) are involved in the regulation of Arabidopsis lateral root development, with *At*SPL10 playing the most dominant role (Yu et al. 2015b). Moreover, the miR156/SPL module has been shown to play a role in lateral root development through its response to growth hormone signals (Yu et al. 2015b), and that plants with reduced miR156 levels exhibited fewer lateral and adventitious roots (Xu et al. 2016). Whereas reduced *SPL12* transcript levels in *SPL12*-RNAi resulted in enhancing alfalfa root regenerative capacity during vegetative propagation, the number of rooted stem propagules was significantly decreased in *35S::SPL12* plants compared with the WT control. The increase in root emergence was observed as early as 13 days after vegetative propagation from stem nodes. Although roots were initiated earlier, this did not result in a significant increase in root length and root biomass in *SPL12*-RNAi genotypes. These results are reminiscent of previous observations by Aung et al. (2015) that while overexpression of miR156 significantly increased root regenerative capacity in alfalfa, the root biomass was not significantly changed during the early stages of root development (3-week-old roots). Taken together, these findings corroborate our results that the miR156-SPL12 module regulates root regeneration capacity at least during the early stages.

Symbiotic nodulation is a complex process between legumes and compatible rhizobia, including the downstream components of signaling pathways that trigger changes in gene expression in both partners. The signals that provide bacterial access to the plant and eventually nodule organogenesis have been well studied in legume species (Mergaert et al. 2020; Roy et al. 2020). miR156/SPL was shown to play a role in nodulation in legume plants. Our previous study found that overexpression of miR156 increased the number of root nodules in alfalfa (Aung et al. 2015). However, the role of miR156/SPL in nodulation may be species-specific, as a reduction in nodulation was reported in other studies for miR156 overexpression plants. For example, when *LjmiR156* was overexpressed in *L. japonicus* it reduced nodule numbers (Wang et al. 2015). *Lj*miR156 also decreased several of early nodulation genes, such as *LjPOLLUX*, *LjCYCLOPS*, *LjNSP1*, *LjNSP2* and *LjNIN* (Wang et al. 2015). Similarly in soybean (*Glycine max*), *Gm*miR156 was found to inhibit nodulation through its negative regulation of *Gm*miR172 (Yan et al. 2013). Wang et al. (2014) showed expression of *GmENOD40* is regulated by *Gm*miR172c, which is activated by *Gm*NINa to control nodule formation upon rhizobial inoculation (Wei et al. 2019). More recently, Yun et al*.* (2022) reported that miR156-SPL9 regulatory system in soybean acts as an upstream master regulator of nodulation by targeting and regulating the transcript levels of nodulation genes in this plant. GmSPL9 is a positive regulator of soybean nodulation which directly binds to the *Gm*miR172c promoter and activates its transcription (Yun et al. 2022). GmSPL9 also directly targets the nodulation master regulator gene, *GmNINa*, and the nodulation marker gene, *GmENOD40*, during nodule formation and development (Yun et al. 2022).

In the current study, we showed that SPL12 has a negative effect on nodulation in alfalfa, as down-regulation of *SPL12* was concomitant with up-regulation of some of the genes known for their involvement in nodulation, including *NIN*, *NSP2*, *IPD3*, *DMI1*, *DMI2*, *DMI3*, *DELLA*, *LysM*, and *CLE13*, with their transcript levels increasing after the 0 dai in alfalfa roots. While overexpression of *SPL12* in alfalfa resulted in reduced nodulation in at least two genotypes (L7 and L5), silencing of this gene (*SPL12*-RNAi) increased nodulation at 14 dai, but by 21 dai there was no exponential increase in the number of nodules in these plants. While a similar finding has not been reported in other plants, this seems to suggest that the onset of nodule development occurs earlier in *SPL12*-RNAi plants compared to WT. It should be noted that the transcript levels of the nodule-related genes were increased in *SPL12*-RNAi at 14 dai but not at 21 dai; a finding that is consistent with the nodule numbers at these two time points. It is also noteworthy that *NIN* transcript levels were increased in *SPL12*-RNAi root at 14 dai, which supports the hypothesis of its possible regulation by SPL12 acting as an essential regulator of nodule organogenesis in legume plants. However, further experiments are required to identify the downstream targets of SPL12 in alfalfa roots.

To balance the costs and benefits associated with root nodule symbiosis and to maintain an optimal number of nodules, plants use the AON pathway (Caetano-Anollés and Gresshoff, 1991); a systemic long-range signaling pathway between roots and shoots. Once nodulation is initiated, two peptides of the CLE family (*Mt*CLE12 and *Mt*CLE13) which inhibit nodulation (Mortier et al. 2010) are normally produced in nodulated roots. These peptides are likely translocated to the shoot (Okamoto et al. 2013), and act through the SUNN receptor, then a shoot-derived inhibitor is delivered to the roots to inhibit nodulation (Mortier et al. 2012). It has been reported that the negative effect of these CLE peptides on nodulation is due to the downregulation of *ENOD11*, an early epidermal infection marker, and NF perception genes (Mortier et al. 2010; Gautrat et al. 2019). Here we showed that the transcript levels of *CLE13* was reduced in *SPL12*-RNAi plants at 14 dai compared to WT, while at 21 dai, *CLE13* was significantly upregulated in the three *SPL12*-RNAi plants. This is consistent with the increased number of nodules at 14 dai and no change at 21 dai, suggesting the potential existence of a regulatory relationship between *SPL12* and *CLE13* on the one hand, and the involvement of SPL12 in the AON symbiotic process in alfalfa on the other.

In *L. japonicas,* the AON *CLE Root Signal* genes (*LjCLE-RS1*) and *LjCLE-RS2* are directly regulated by *Lj*NIN (Soyano et al. 2014). Wei et al. (2019), reported that the AON soybean *Rhizobia-Induced CLE1* (*GmRIC1*) and *GmRIC2* (orthologous to *LjCLE-RS1* and *LjCLE-RS2*) are directly activated by *Gm*miR172c, which is transcriptionally activated by *Gm*NINa, leading to the activation of the AON pathway. Yun et al*.* (2022) showed that miR156b-GmSPL9d module acts as an upstream master regulator of nodulation by regulating *GmNIN* and *GmmiR172* in soybean. The highly conserved regulatory role of miR156 in nodulation and the observed increase in nodule number in *SPL12*_RNAi plants and the altered *CLE13* transcript levels, led us to propose that miR156-SPL12 module may regulate nodulation through its involvement in the AON pathway in alfalfa symbiosis. However, further experimentation is needed to substantiate this hypothesis, as AON is a complex process and there are multiple genes involved in this pathway (reviewd by Roy et al. 2020).

Aung et al. (2017) reported that overexpression of alfalfa’s miR156 increased nodule numbers, nitrogenase activity, and the transcript levels of bacterial genes *FixK* (providing activation of nodule respiration), *NifA* (nitrogenase-encoding) and *RpoH* (sigma 32 factor for effective nodulation) in alfalfa roots inoculated with *S. meliloti*. Similarly, our study indicated that at 14 dai, silencing *SPL12* stimulates nitrogenase activity in RNAi12-7 and RNAi12-29. RT-qPCR transcript analysis also showed that silencing of *SPL12* enhanced the transcript levels of *S. meliloti*’s *RpoH*, *FixK* and *nifA* gens in alfalfa. Although, it is estimated that mature alfalfa plants could obtain up to 80% of their total nitrogen requirements through biological fixation (Provorov and Tikhonovich 2003), emerging seedlings and those grown under abiotic stress conditions, which are known to reduce biological nitrogen fixation (Miransari and Smith 2007), still require nitrogen fertilizers, and thus enhancing nodulation and nitrogen fixation at the early stages of plant development should have agronomic and economic benefits to farmers.

In a previous transcriptomic analysis, both *SPL12* and *AGL6* were shown to be downregulated in roots of miR156OE alfalfa (Gao et al. 2016; Aung et al. 2017). In the current study, the highest *AGL6* transcript levels were detected in roots of *SPL12* overexpression genotypes, and further analysis revealed that *AGL6* was under the regulation of SPL12. *AGL6* belongs to the MADS-box protein family that includes transcription factors with the conserved MADS-box domain (Shore and Sharrocks 1995; Theißen and Gramzow 2016). Dong et al. (2021) recently identified 120 *MsMADS-box* genes in the alfalfa genome (designated as *MsMADS001* to *MsMADS120*), with *AGL6* corresponding to *MsMADS010*.

*AGL6* in alfalfa is an orthologous to *AtAGL79* in Arabidopsis, that is regulated by *At*SPL10 and is involved in regulating lateral root development through the miR156-SPL pathway (Gao et al. 2018b). Although the MADS-box genes have been well characterized in many plants (Puig et al. 2013; Schilling et al. 2018; Zhang et al. 2019), information on their role in regulating legume-rhizobia interactions is still in its infancy. In soybean, a MADS-box protein, *Gm*NMHC5, positively regulates root development and nodulation (Liu et al. 2015), while *Gm*NMH7 is a negative regulator of nodulation (Wei et al. 2019). In common bean (*Phaseolus vulgaris*) *Pv*AGLs have been proposed as new protagonists in the regulation of nodulation (Íñiguez et al. 2015). Ayra et al. (2021) recently reported that *PvAGL*-RNAi plants in common bean showed decreased rhizobial infection as well as decreased transcript levels of some of the early nodulation genes like *PvNIN*. They also produced more small and ineffective nodules indicating an alteration in the AON pathway (Ayra et al. 2021). Our finding that *SPL12*-RNAi and *AGL*6-RNAi had increased nodulation indicates that SPL12 controls nodulation in alfalfa by directly regulating *AGL*6, and that SPL12 and AGL6 are possibly involved in AON pathway in this plant.

## Conclusion

Based on previous reports on the role of miR156 in the regulation of nodulation, nitrogen fixation and root regenerative capacity in alfalfa (Aung et al. 2015), the current characterized the function of SPL12, as a target of miR156, in the aforementioned traits. We found an enhancement in alfalfa root regenerative capacity during vegetative propagation in *SPL12*-RNAi plants. In addition, we revealed that SPL12 has a negative effect on nodulation, as silencing of *SPL12* improved nodulation and nitrogen fixation in alfalfa.

We also determined direct binding of SPL12 to the *AGL6* promoter. Our findings that SPL12 directly regulates this gene suggest that the miR156/SPL12 regulatory pathway is involved in regulating nodulation by directly targeting and activating the transcript levels of *AGL6* in alfalfa. Taken together, SPL12 along with *AGL6* modulate alfalfa nodulation and nitrogen fixation.

## Supplementary Information

Below is the link to the electronic supplementary material.

Supplementary file1 (PDF 1389 kb)

Supplementary file2 (XLS 37 kb)

Supplementary file3 (PDF 132 kb)

Supplementary file4 (PDF 18 kb)

Supplementary file5 (XLSX 291 kb)

Supplementary file6 (XLSX 82 kb)

Supplementary file7 (XLSX 19 kb)
