# Supplementary material for: Deciphering the role of SPL12 and AGL6 from a genetic module that functions in nodulation and root regeneration in Medicago sativa
Source: Plant Mol Biol. 2022 Aug 17;110(6):511–29. doi: 10.1007/s11103-022-01303-7 (PMC9684250; doi:10.1007/s11103-022-01303-7)
Supplement: Supplementary file 1 — Supplementary file1 (PDF 1389 kb) [file 11103_2022_1303_MOESM1_ESM.pdf]

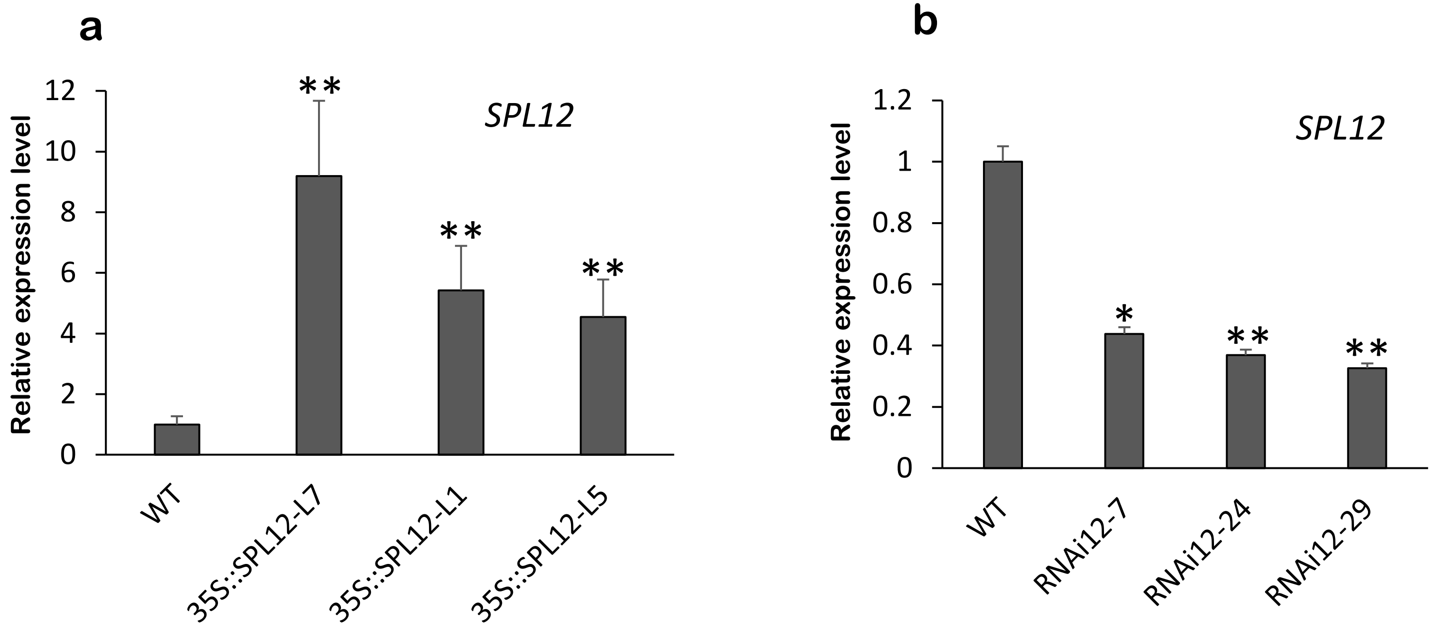


**Fig. S1 Transcript analysis of *SPL12* gene in different alfalfa genotypes.**

A) Relative *SPL12* transcript levels in *35S::SPL12* plants (n = 3). B) Relative *SPL12* transcript in *SPL12*-RNAi plants (n = 3). * and ** indicate significant differences relative to wild type using *t* test $p < 0.05$, $p < 0.01$, respectively. Error bar indicates standard deviation.

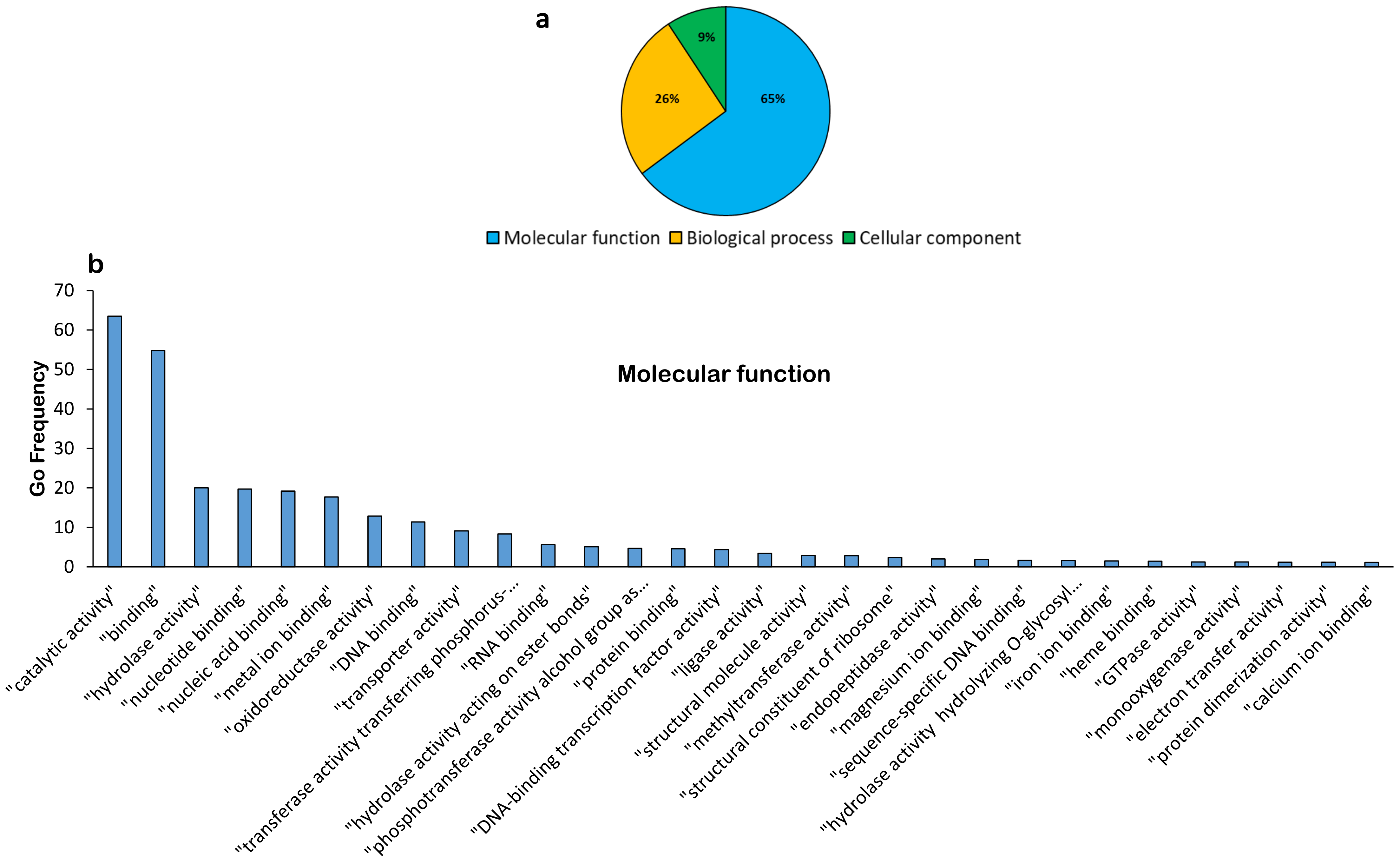

a
9%
26%
65%
Molecular function
Biological process
Cellular component
b
Go Frequency
Molecular function
70
60
50
40
30
20
10
0
"catalytic activity"
"binding"
"hydrolase activity"
"nucleotide binding"
"nucleic acid binding"
"metal ion binding"
"oxidoreductase activity"
"DNA binding"
"transporter activity"
"transferase activity transferring phosphorus-...
"RNA binding"
"hydrolase activity acting on ester bonds"
"phosphotransferase activity alcohol group as...
"protein binding"
"DNA-binding transcription factor activity"
"ligase activity"
"structural molecule activity"
"methyltransferase activity"
"structural constituent of ribosome"
"endopeptidase activity"
"magnesium ion binding"
"sequence-specific DNA binding"
"hydrolase activity hydrolyzing O-glycosyl...
"iron ion binding"
"heme binding"
"GTPase activity"
"monooxygenase activity"
"electron transfer activity"
"protein dimerization activity"
"calcium ion binding"

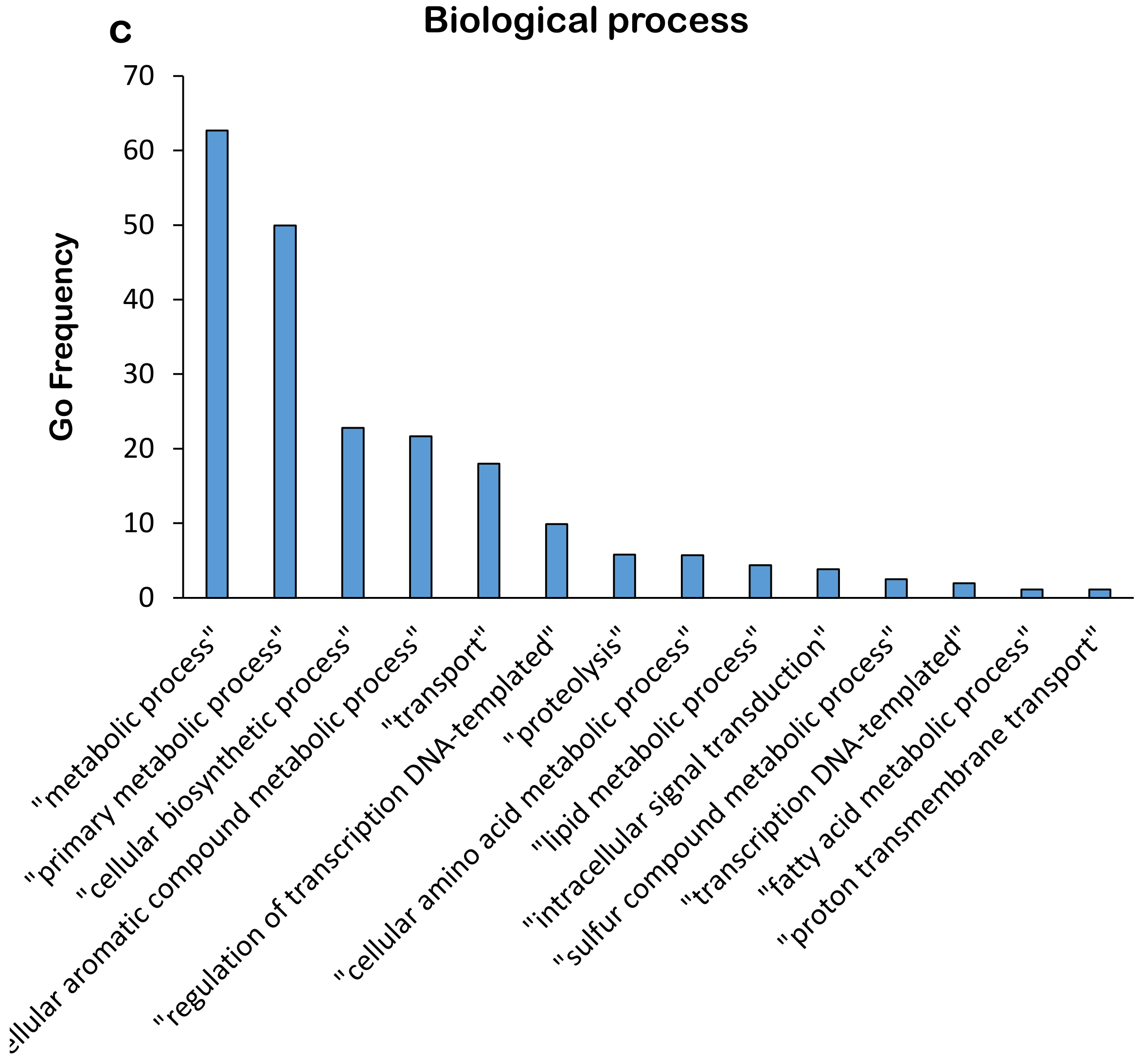
c
Biological process
Go Frequency
70
60
50
40
30
20
10
0
"metabolic process"
"primary metabolic process"
"cellular biosynthetic process"
ellular aromatic compound metabolic process"
"transport"
"regulation of transcription DNA-templated"
"proteolysis"
"cellular amino acid metabolic process"
"lipid metabolic process"
"intracellular signal transduction"
"sulfur compound metabolic process"
"transcription DNA-templated"
"fatty acid metabolic process"
"proton transmembrane transport"

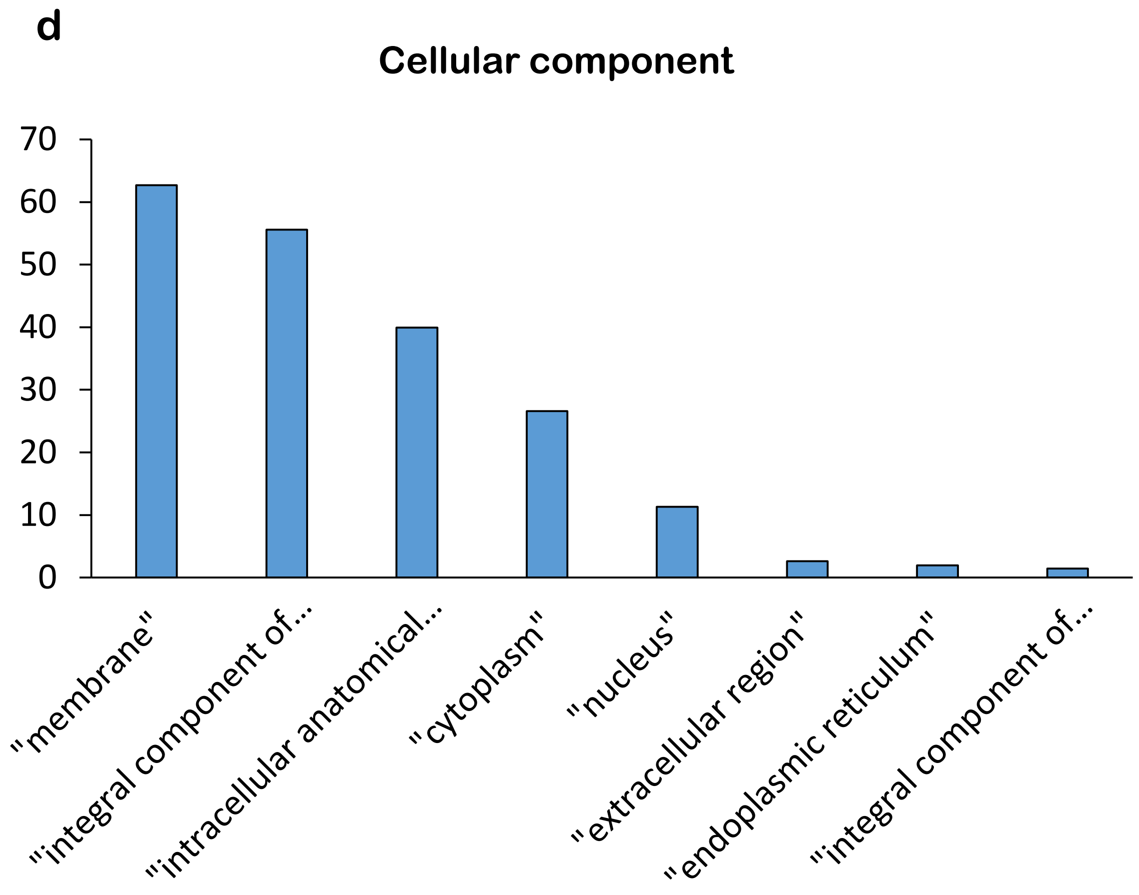
d
Cellular component
70
60
50
40
30
20
10
0
"membrane"
"integral component of...
"intracellular anatomical...
"cytoplasm"
"nucleus"
"extracellular region"
"endoplasmic reticulum"
"integral component of...

**Fig. S2 Gene Ontology (GO) enrichment analysis of DEGs between *SPL12*-RNAi and WT**

A) Gene Ontology (GO-term) –based percent representation of DEGs between WT and *SPL12*-RNAi in alfalfa roots in cellular components, biological process, and molecular functions. Go frequency of B) molecular function, C) cellular component and D) biological process.

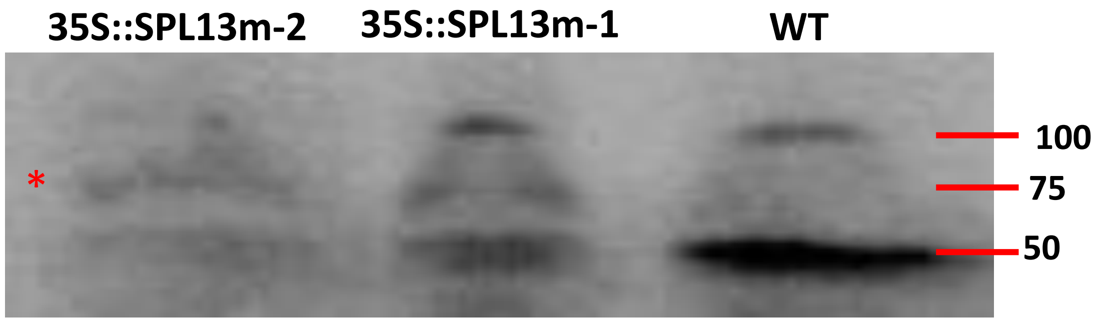


**Fig. S3 Detection of SPL12m-GFP fusion protein in putative alfalfa transgenic plants using Western blotting.**

CTTTCTGTGTTATGTGATGCTGAGGTTGCTCTTATCATTTTCTCCGGTCTTGGCAAGCTTTTTCAATACAGTACCACAGAGTGAGCAATTCATTTTCTTT
TAATATATATATCTTTAAGCAATTTTTTATCTCTTCGTTAGTTTTTTAAATACAAGCTTGGTCATAATTAAAAATATTCTAGGGGTTGTAAAATTATCTGTT
TAGCGCATTTTTGTTTGCCAAGAGAAATATCATAAGGTAAAAGCTACCGGCCGGTGGATTGGTCATTCATGAAGACAACTTGTTTGGCAAAGCACAA
CCACTTGATAGTTCTAGCAACATTCACTCATCATTTGACTAAGTTATTCCTTCTTGTCTTGGTTATAAGTAAACTAATTTCACACATTAAGAAATTCAGTA
AATATAATTAATCTTCTTGCAACAAAAAAAATGGTACCCATCATAAAAATTTGTTTCATAAGAATGAAATGTCACTAAAAAAATAAAATATATATAAATTA
AAGAGTATTTTAGGATATGAGTCTTTATATCAAAAGTAGTCATATTAGCTTAATTATATTTTAAAAAAGTATGCACTTTTTTTTTTTTTTATCTTTATTTGA
GATTGGAATTAGGGGTGTCAATTTCATCTCTGAATGGGGATCTAAGCGGGTACCATCAAATTTGAAGTTACGTAGACGGAGAATTTTTTTCTATTGG
GGATGGGGGAGAAAGTATTCTCTGATAATAGTGCAGGGACACGATCATACCCCCCGATACATGGAGACTCGTCCCCGAGCAATTCAGTATAATATATT
TTATATTGGTATATACAAATATTTCCTATGTTATATTTATTTATCATTTTACGCAATTTTTTTATATGTAATGTTTACTGTAATGCCTTAATTATATTATATTTGT
TTTAATGTTAAATTTTAATTTTTTTAAAATAATATAAATTTAGTTATTTTTACATGCCGATGGATCTCTGTGGGACCGTGGTGATGTGCGGGGGTGAGG
ACAGGGAGGAAAAACTCTCCAAGACGGGGAATGGGGATGAGGGACAATTTAAGTGGCGGGAGAGGAAACATAGATGTACTCCTTGTCTCCTCCC
CACCCCATCCATTGACATCCCTGACTAGAATAGCTAGTAGAGTATTTTTTTTTTTTTTTGACAAAAAACAAGTAGAGTACGATAAAAAGGATTATTTTA
GTCATGGATTGAACTATAATATTCCAAGATAATTTGACCTTAATGAAGTTTTACTAACTACTTAAACTCAACCATTTAGTTAGACTTTAACATCAAAATG
TGTTTGAGTTGTGTAAGTTTGAGGTGGATTTGTGGGAGTAACTTGATAGCGGTTATAAAATTAAATTTGAGTTTCTTACAAATTTGTCATTGGAGATG
TTCTAAGGATCATACTAGCTTATATTCTAAGAGCACATGTTAAAGATTTTTACCAATAGTAATTATAATTGAAAAAAGTAATTTTCAACTTTTTGAAGAA
TGTACAAAGTTTTCAATAAGAAATTTCTATATTTAATGCACTAACAAGTGCCTTAACTATAAGGGTGCATTTTAACATTCCTCTTGTTGTAATACAAACA
ATGATGTTAGTTTTATTCAACGGGAGAGAATGTAGTTGTTAGTCCCTATATTTTTATAACGTATTATCTTTTTTTTTTTTGTTTCACGGTTTATTTCAATAAT
TTAACTCAAGTCAAAATTATATGGTACTAACCAATATAATTGATATACCTTGTTCATTCTGATCAGCTTGAACAAAATCATTGAGAAGTATCGTCAATGTT
GCTTCAACAATATGTCTGAGAATGGTGACTTAGGAGAACATGAGTCACAGG

**Fig. S4 Promoter sequence of the alfalfa *AGL6* gene with putative SBD binding elements.**

Nucleotides highlighted with yellow and blue colors represent putative SBD binding motifs with ‘GTAC’ core sequences and forward and reverse primer sequences used for ChIP-qPCR, respectively. The red text shows coding sequences of *AGL6*.
