# Supplementary material for: Deciphering the role of SPL12 and AGL6 from a genetic module that functions in nodulation and root regeneration in Medicago sativa
Source: Plant Mol Biol. 2022 Aug 17;110(6):511–29. doi: 10.1007/s11103-022-01303-7 (PMC9684250; doi:10.1007/s11103-022-01303-7)
Supplement: Supplementary file 3 — Supplementary file3 (PDF 132 kb) [file 11103_2022_1303_MOESM3_ESM.pdf]

Deciphering the role of SPL12 and AGL6 from a genetic module that functions in nodulation and root regeneration in *Medicago sativa*

Journal: Plant Molecular Biology

Vida Nasrollahi [1,2]. Ze-Chun Yuan [1]. Qing Shi Mimmie Lu[1]. Tim McDowell [1]. Susanne E. Kohalmi[2]. Abdelali Hannoufa[1,2,*]

[1] Agriculture and Agri-Food Canada, 1391 Sandford Street, London, Ontario, N5V 4T3, Canada

[2] Department of Biology, University of Western Ontario, 1151 Richmond Street, London, Ontario, N6A 3K7, Canada

[*]**Author for correspondence**: **Email**: Abdelali.Hannoufa@agr.gc.ca

Table S2: Buffers used in ChIP assay and their components

Buffers	Chemicals	Concentration	Buffers	Chemicals	Concentration
Extraction buffer 1	Sucrose	0.4 M	Extraction buffer 2	Sucrose	0.25 M
	Tris-HCl (pH=8)	10 mM		Tris-HCl (pH=8)	10 mM
	$MgCl_2$	10 mM		$MgCl_2$	10 mM
	β-ME	5 mM		Triton X-100	1%
	PMSF	0.1 mM		β-ME	5 mM
	Protease inhibitor[1]	2 tablets/ 100mL		PMSF	0.1 mM
				Protease inhibitor[1]	1 tablet/10mL
Extraction buffer 3	Sucrose	1.7 M	Nuclei lysis buffer	Tris-HCl (pH=8)	50 mM
	Tris-HCl (pH=8)	10 mM		EDTA	10 mM
	$MgCl_2$	2 mM		SDS	1%
	Triton X-100	0.15%		Protease inhibitor[1]	1 mini tablet/10mL
	β-ME	5 mM	ChIP dilution buffer	Triton X-100	1.10%
	PMSF	0.1 mM		EDTA	1.2 mM
	Protease inhibitor[1]	1 mini tablet/10mL		Tris-HCl (pH=8)	16.7 mM
	Sucrose	1.7 M		NaCl	167 mM
Elution buffer	SDS	1%	High salt wash	SDS	0.10%

	$NaHCO_3$	0.1M	buffer	Triton X-100	1%
Low salt wash buffer	SDS	0.10%		EDTA	2 mM
	Triton X-100	1%		Tris-HCl pH=8)	20 mM
	EDTA	2 mM		NaCl	500 mM
	Tris-HCl (pH=8)	20 mM	LiCl wash buffer	LiCl	0.25 M
	NaCl	150 mM		IGEPAL-CA630	1%
				Deoxycholic acid	1%
TE buffer	EDTA	1 mM		EDTA	1 mM
	Tris-HCl (pH=8)	10 mM		Tris-HCl (pH=8)	10 mM

[1] Obtained from Sigma-Aldrich, Canada
