# Supplementary material for: Deciphering the role of SPL12 and AGL6 from a genetic module that functions in nodulation and root regeneration in Medicago sativa
Source: Plant Mol Biol. 2022 Aug 17;110(6):511–29. doi: 10.1007/s11103-022-01303-7 (PMC9684250; doi:10.1007/s11103-022-01303-7)
Supplement: Supplementary file 4 — Supplementary file4 (PDF 18 kb) [file 11103_2022_1303_MOESM4_ESM.pdf]

Deciphering the role of SPL12 and AGL6 from a genetic module that functions in nodulation and root regeneration in *Medicago sativa*

Journal: Plant Molecular Biology

Vida Nasrollahi [1,2]. Ze-Chun Yuan [1]. Qing Shi Mimmie Lu[1]. Tim McDowell [1]. Susanne E. Kohalmi[2]. Abdelali Hannoufa[1,2,*]

[1] Agriculture and Agri-Food Canada, 1391 Sandford Street, London, Ontario, N5V 4T3, Canada

[2] Department of Biology, University of Western Ontario, 1151 Richmond Street, London, Ontario, N6A 3K7, Canada

[*]**Author for correspondence**: **Email**: Abdelali.Hannoufa@agr.gc.ca

Table S3. Validation of RNA-Seq data using qRT-PCR

Gene name	RNAi12-24/WT		RNAi12-29/WT	
	RNA-Seq	qRT-PCR	RNA-Seq	qRT-PCR
ABA/WDS induced protein	--	0.73	0.07	0.88
GRAS family transcription factor	0.53	0.53	0.41	0.28
WRKY family transcription factor 41	0.2	0.7	0.27	0.51
lateral organ boundaries (LOB) domain protein	0.57	0.89	0.47	0.23
Nod26 MIP family transporter	5.52	1.56	3.57	1.27
peptide/nitrate transporter	3.98	1.53	2.14	2.33
two-component response regulator ARR3-like protein	2.52	77.39	3.85	4.39
sulfate/bicarbonate/oxalate exchanger and transporter sat-1	4.53	2.22	2.54	1.81
high affinity sulfate transporter type 1	4.37	4.9	1.87	2.1
nitrate reductase [NADH]-like protein 1	8.15	1.24	2.50	1.09
nitrate reductase [NADH]-like protein 2	6.29	4.14	1.99	1.01
high affinity sulfate transporter type 1	1.95	2.36	1.74	2.78
component of high affinity nitrate transporter	2.02	0.43	1.52	0.77
caffeic acid O-methyltransferase	6.52	2.96	5.12	3.12
